# Working on mental health stigma in education: a multicentre community-based clinical trial

**DOI:** 10.3389/fpubh.2025.1515444

**Published:** 2025-06-27

**Authors:** Lucía Santonja-Ayuso, Laura Andreu-Pejó, José Vicente Carmona-Simarro

**Affiliations:** ^1^Nursing Department, University Jaume I, Castellón, Spain; ^2^Nursing Department, European University of Valencia, Valencia, Spain

**Keywords:** high school teachers, education, mental health, stigma, psychiatric nursing

## Abstract

**Background:**

High school teachers have a key role to play in supporting their students in this stage of adolescent growth, especially given the increasing prevalence of mental disorders in this population. However, it has been observed that these teachers lack confidence, commitment and knowledge related to the management of mental health problems and show a high level of stigma. The goal of this research was therefore to evaluate the effectiveness of a mental health prevention and promotion training programme by led mental health nurse, aimed at reducing the stigmatising attitudes of teachers at schools in Spain.

**Methods:**

A multicentre, non-randomised, community-based study was therefore carried out, with a pre-post-test and a three-month follow-up. The teachers (*n* = 169) were divided into the experimental group (*n* = 103) and the control group (*n* = 66). The training programme was conducted over different 4 different sessions (8 h in total) and the dependent variable was the Stigma Attribution Scale.

**Results:**

Statistical analyses showed a significant improvement in the level of stigma at the post-programme evaluation for all scale dimensions except Responsibility and Help, which were also maintained over time, since at 3 months the reduction in the level of teacher stigma was lower for all scale dimensions except Responsibility and Avoidance. The effect size of the improvement in stigma level was moderate-high for Pity, Coercion and Segregation (*d* ≥ 0.5).

**Conclusion:**

This training programme based on anti-stigma measures led by a mental health nurse was effective in reducing stigmatising attitudes in high school teachers in both the short and the long term.

**Clinical trial registration:**

https://doi.org/10.1186/ISRCTN63945853, identifier ISRCTN63945853.

## 1 Introduction

According to the World Health Organization (WHO), one in seven adolescents worldwide aged 10–19 has a mental health problem; emotional disorders, eating disorders and psychosis are the most prevalent (1). In Spain, the percentage of adolescents with a diagnosed psychopathological disorder is 20.8% (21.4% girls and 20.4% boys), which places it at the top of the European countries with the highest prevalence of mental health disorders in this type of population (2). This increase in prevalence from previous years may be related to the context and restrictive protective measures that were put in place in the Covid-19 pandemic years (3, 4). In this regard, evidence has shown that such restrictions appeared to predispose people to anxiety, social isolation and depressive symptoms, amongst others (5, 6), facilitating the emergence of new cases of adolescents with mental health problems, aggravating the situation of those most vulnerable and leading to negative consequences that could persist in the long term (7–9).

This is why national and international institutions, such as the Spanish Ministry of Health and the WHO, have emphasised the importance of implementing community-based interventions (1, 10). These interventions should focus on the prevention and promotion of mental health amongst adolescents, particularly within the educational setting. Schools represent an ideal environment to reach a large number of adolescents, as they spend considerable time there, and teachers accompany them throughout the day (11, 12). Therefore, a key competency for teachers is to acquire knowledge and develop positive attitudes regarding mental health. This enables teachers to serve as role models for adolescents within the classroom, encouraging them to seek help and supporting their growth and needs related to mental health (13, 14).

However, in order to become support agents, teachers have to be given appropriate training in mental health (15). In this regard, recent research has not only shown that teachers’ knowledge and training in mental health is low (15–17), but they also have other limitations related to skills shortages, such as lack of confidence in their performance and lack of engagement with their students (17–20). Stigma is also a major barrier to addressing mental health problems in general and, in school settings, contributes to social isolation, minimisation of symptoms and poorer management of the consequences of mental health disorder (21).

It is also important to take into account that in Spain, teachers do not include in their curricula systematic training in mental health that includes the recognition and early detection of the most prevalent mental disorders in adolescence, the identification and management of risk and protective factors for mental health in the classroom, active help-seeking (22, 23) or anti-stigma interventions (21, 24, 25).

Therefore, an evident need is recognised for the implementation of mental health training programmes for secondary school teachers, which include the reduction of stigmatising attitudes and beliefs (14, 15, 18). Within this context, it has been deemed appropriate for the application and leadership of such interventions to be undertaken by a specialised mental health nurse, given that in Spain, upon completing a regulated two-year training programme, these professionals are qualified to carry out prevention and promotion interventions related to mental health in both clinical and community settings, targeting the general population or specific groups (26–28).

In addition, based on a pilot study in which we provided an anti-stigma training programme to teachers (29), and in view of the promising results obtained, this research aims to evaluate the effectiveness of a training programme in mental health prevention and promotion, taught by a mental health nurse, in order to reduce stigmatizing attitudes (Anti-stigma training program) amongst teachers of different institutes in the Valencian Community, Spain.

## 2 Materials and methods

### 2.1 Study design

A multicentre community-based non-randomised clinical trial was conducted to evaluate the effect of a mental health nurse-led anti-stigma training programme based on the prevention and promotion of mental health and focused on the reduction of stigmatising attitudes amongst high school (IES in Spanish) teachers in the self-governing region of Valencia (Spain). In order to reduce systematic errors and provide quality results, the TREND checklist was used for reporting and publishing (30).

### 2.2 Study participants

Based on accessibility, sectorisation, youth population density and the approximate number of teachers per school that formed part of the Health Department in the geographical area where the study was carried out, a sample calculation The parameters used for the sample calculation were based on statistical conventions widely accepted in research, supported by the methodological literature (31, 32): a 95% confidence level, a magnitude of effect of 0.50 (which was classified as moderate according to the Cohen scale since; it indicates that the difference between groups is equivalent to 0.5 standard deviations, enough to be perceptible but not extreme), a statistical power of 0.80 and alpha of 0.05. In order for the sample to be representative and appropriate, and for the error margin to be minimal, a total of 80 participants in all were required.

First, legal and complete information (including informed consent) was sent to all schools that were part of the principal investigator’s health department and that met the inclusion criteria (*N* = 12 schools) (Table 1).

**Table 1:** Eligibility criteria for the sample.

Type of entity	Inclusion criteria	Exclusion criteria
Participants	Working full time at the school at the time of submission of the programme. Spanish as native language or official accreditation of a high level of Spanish (C1^†^). Willingness to attend 100% of the sessions. Signing the informed consent form.	Working part-time and/or being on leave of absence and/or sick leave. Showing conditions that make it impossible to answer the questionnaire (motor, hearing, visual or speech functional diversity). Previous participation in a similar programme.
Educational establishments	Public or private. Compulsory Secondary Education and/or Baccalaureate (from the age of 12).	Special education schools. Schools whose official language is not Spanish/Valencian.

C1^†^, operational and effective language level according to the Spanish Ministry of Education and Vocational Training.

Secondly, and after the acceptance of three schools, an informative meeting was arranged with the teachers of each of them to explain the intervention and give them the informed consent, which had to be signed before participating. It was decided to carry out the study at high schools given that in this environment we work with adolescents from 12 to 18 years of age; this was the age range chosen as it is the period of onset of most mental health problems in adolescence and adulthood (1).

**Figure 1:**
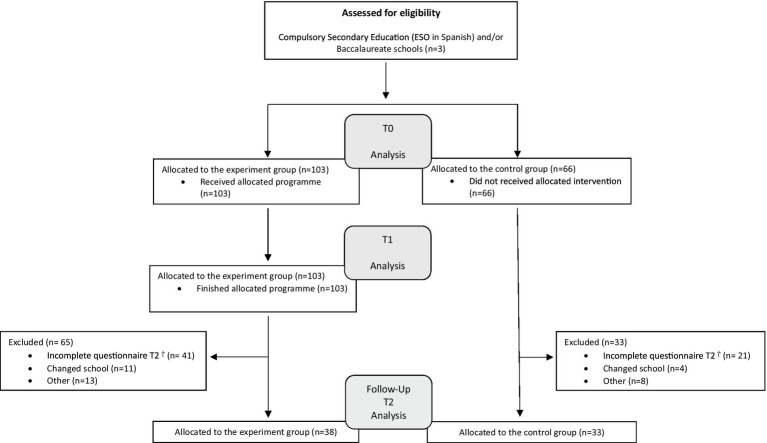
Consort flow diagram illustrating the recruitment and participation process.

Finally, a total of 169 participants were obtained. However, given the reported results and the limited participation in a previous pilot programme carried out by this research group (29), and with the intention of obtaining an experimental group and a control group, two groups were carried out through intentional sampling according to the feasibility of adequately completing the training programme and the possibility of effective follow-up of the cohort (Figure 1). The recruitment took place during the months of May and June 2023 and the control group was given the opportunity to participate in a second edition of the anti-stigma training program.

### 2.3 Anti-stigma training programme

The anti-stigma training programme was developed based on the available scientific evidence and the content of guides, manuals and recommendations of both experts and prestigious national and international entities in the field of mental health (9, 10, 33).

The content of the programme focused on training on the risk and protective factors present in the adolescent period, the reduction of stigmatising beliefs, the definition of the role that teachers should play in this area, and training on the algorithm of action and the social and health resources available in the region. The anti-stigma programme was evaluated by professionals in the field of psychiatry and university lecturers and its usefulness was assessed in a previous pilot study, reporting favourable results (29). On the request of the teachers taking part in the pilot study, and after checking with the available literature (34–36), a block of content related to emotions and healthy emotional regulation was added to the programme.

The intervention was thus composed of four different thematic blocks and divided into 4 different sessions (Table 2). In each session, the contents were shown by means of a digital presentation and were complemented with methods and techniques of group education (37, 38) (Table 2). References to any type of diagnostic entity were made following the criteria of the Diagnostic and Statistical Manual of Mental Disorders-5 (39).

**Table 2:** Anti-stigma management programme composition.

Content		Methodology and dynamics			
		Classroom research techniques (expressing your ideas, previous experience)	Expository techniques (reorganising information)	Analysis techniques (reflecting)	Skills development techniques
Session 1					
Prevention and promotion of Mental Health in adolescence	Defining the concept of mental health in adolescence. Being familiar with the biopsychosocial changes of development in the adolescent stage. Risk and protective factors in the classroom. Most prevalent mental health problems in the adolescent stage. General early symptomatology. Consumption of toxic substances.	Questionnaire Brainstorming	Talk-colloquium Participative lesson		
Session 2					
Stigma and Psychoeducation in Psychosis	Concept of stigma related to mental health. Strategies to eradicate stigma from the classroom. Useful online resources to work and reduce stigma in the classroom. Myths and realities: psychosis. Stress vulnerability model. Early detection.	Questionnaire Brainstorming PhotoWord: *Mañana puedes ser tú* (68)	Talk-colloquium *Lo Hablamos* (60) Video with discussion	Video of a person with schizoaffective disorder recounting his experience at the institute.	Operational simulation
Session 3					
Emotions and Healthy Emotional Regulation	Myths about emotions. Concept of emotion and main functions of basic emotions. Distinguishing between simple and complex emotions. Identifying when emotions are not very adaptive. Being familiar with the abbreviated outline of healthy emotional regulation (distancing from emotion, identification and communication, distraction and healthy coping) and adapted its to the school context from the perspective of mental health promotion.	Questionnaire Brainstorming	Participative lesson	Case Discussion	Operational simulation
Session 4					
Algorithm for action and referral to the health system. Community resources	Summary of the above points. Presenting the algorithm for referral to health services. Being familiar with other community resources.	Questionnaire Brainstorming	Talk-colloquium		

The anti-stigma training programme was delivered by a mental health nurse, in a face-to-face format over 4 different sessions (each lasting 2 h) in consecutive weeks. All the sessions were held at the schools themselves in teachers’ working hours in July 2023, as the school term was over, and teachers had more time available. There were no financial incentives or incentives of any kind for the teachers taking part, and there were no adverse effects during the programme or after it finished.

### 2.4 Instruments of measurement

An *ad hoc* questionnaire was drawn up to collect the socio-demographic data of the participating teachers (gender, age, number of children, work experience, place of residence, level of studies), and a set of variables to determine their experience with students with mental health problems (tutoring students, familiarity with mental health services, familiarity with self-harm and/or suicidal behaviour, crisis care and perceived feelings during care).

The Attribution Questionnaire-27 (AQ-27) scale was used to assess the level of stigma, as designed by Weiner et al. (40) and validated in Spain by Muñoz et al. (41). This scale has a Cronbach’s alpha close to 0.855, showing appropriate psychometric properties for this population (41). It is a 27-item scale, which assesses nine different constructs: Responsibility (believing that people with a mental disorder are responsible for their condition), Pity (feeling sorry for a person because they have a mental disorder), Anger (feeling anger towards people with a mental disorder), Danger (believing they are dangerous), Fear (feeling afraid to be around them), Help (believing they are dependent and need help), Coercion (forcing them to comply with external decisions because they believe they are incapable), Segregation (they should live apart from the community) and Avoidance (avoiding contact), using a 9-point Likert-type scale (from 1 “not at all” to 9 “very much”). The scores for each factor are obtained by adding up three specific items; the total score for each of the nine items ranges from 3 to 27. The score is inverted before adding up the factors in the case of the Avoidance dimension. The higher the score, the higher the stigma attribution.

### 2.5 Data collection and analyses

The experimental group answered the *ad hoc* questionnaire and the AQ-27 scale before the training programme, in July (T0). Subsequently, they also responded to AQ-27 at the end of the programme (4 weeks later, T1) and at 3 months follow-up, in October (T2). The control group only answered the questionnaires two times: before the experimental group completed the programme in July (T0) and 5 months after, in October (T2). Subsequently, the control group was given the opportunity to take the program.

All responses were anonymous and were coded and recorded in a computer programme. To link the pre (T0)- and post (T1)- and follow-up (T2) questionnaires, participants had to identify their questionnaire with their initials and the last two digits of their telephone number. The information was stored and kept by the health department. The extraction, analysis and production of the final report lasted until December 2023.

It was an open trial as there was no possibility of blinding of participants or the principal investigator, as she was responsible for conducting the intervention programme. However, the researcher in charge of the statistical analysis did not know whether the data came from the intervention or control group in T0 and T2.

Regarding bivariate analysis, a 95% CI was considered for a *p-value* of <0.05. Parametric tests were used since the variables followed a normal (Gaussian) distribution. First, a univariate descriptive analysis was performed, and the Chi squared test statistic was used to determine the relationship between sex and other categorical variables of interest.

Secondly, to assess the efficacy of the intervention in the experimental group, we adapted the results of the AQ-27 pre-post programme and follow-up (T0, T1 and T2), with the aim of assessing the existence of statistically significant differences between the three moments, using a Student’s t-test. In the control group, statistically significant differences between T0 and T2 were analysed. In this way, not only was it assessed whether the intervention had been valid, but also how the controls behaved without intervention.

To quantify the magnitude of the differences in the AQ-27 dimension scores between the participating teachers who undertook the anti-stigma programme (experimental group) and those who did not (control group), Cohen’s d was calculated. The statistical programme ISBM SPPS version 22® was used for the statistical analysis.

### 2.6 Ethical considerations

At the three schools in the study, the programme protocol was explained. Both the school management and the teachers who took part did so voluntarily. The privacy and confidentiality of both their personal data and the answers to the questionnaires was guaranteed.

This intervention was approved by the Research Ethics Committee in accordance with the ethical standards of the 1964 Helsinki declaration and its subsequent amendments or comparable ethical standards and has been registered with ISRCTN (ID: ISRCTN63945853).

## 3 Results

### 3.1 Socio-demographic profile of the sample

The experimental group sample consisted of 103 participants, 68.90% (*n* = 71) of whom were female and 31.10% (*n* = 32) male. The mean age was 46.82 ± 9.98 years; 47.21 ± 9.88 years in females and 45.94 ± 10.30 years in males. 80.60% (*n* = 83) of participants resided in urban areas and had one child on average and 79.60% (*n* = 82) of the participating teachers had an academic level of Bachelor’s or Associate degree. The mean number of years of teaching experience was 17.01 ± 9.88 years (Table 3).

**Table 3:** Sociodemographic profile of the sample.

Sociodemographic variables	Experimental group		Control group		Total	*p*-value
	AF	RF (%)	AF	RF (%)	AF	
Sex						0.476
Man	32	31.10	24	36.40	56	
Woman	71	68.90	42	63.60	113	
Place of residence						0.104
Urban	83	80.60	46	69.70	129	
Rural	20	19.40	20	30.40	40	
Level of education						0.290
Doctorate	2	1.90	1	1.52	3	
Master	19	18.40	19	24.24	38	
Bachelor’s degree	82	79.60	46	69.70	128	

Sociodemographic variables	Experimental group	Control group	Total	*p*-value
	Mean (SD)	Mean (SD)	Mean (SD)	
Children	1.37 (0.95)	1 (0.89)	1.22 (0.94)	0.024
Age	46.82 (9.98)	46.33 (11.45)	46.63 (10.55)	0.055
Woman	47.21 (9.88)	47.43 (11.24)		
Man	45.94 (10.30)	44.42 (11.81)		
Work experience in years	17.01 (9.88)	15.61 (11.22)	16.46 (10.41)	0.320

*p*-value obtained from the Chi-square test; AF, absolute frequency; RF, relative frequency; SD, Standard Deviation.

The control group sample consisted of 66 teachers, who did not take part in the intervention. 36.4% (*n* = 24) were male compared to 63.60% (*n* = 42) female. The mean age was 46.33 ± 11.45 years, with one child. 69.70% (*n* = 46) were urban residents. The mean number of years of teaching experience was 15.61 ± 11.22 years and the academic level of 69.70% (*n* = 46) was Degrees.

Regarding the specific variables related to teaching experience, 52.66% (*n* = 89) were tutors. 52.66% (*n* = 89) stated that they did not know any students under follow-up in specialised mental health care. 53.85% (*n* = 91) did not know of any students who self-harm and 52.66% (*n* = 89) said they were unaware of any cases of suicide attempts. 52.66% (*n* = 89) said that they had had to act in case of a student’s crisis, where 20.71% (*N* = 35) of those who responded affirmatively reported having felt “bad” when doing so (Supplementary Table 1).

No statistically significant differences were found between the experimental group and the control group in terms of socio-demographic characteristics (except in the number of children, *p*-value<0.05) and with respect to the specific variables related to teachers’ experience. The results showed that there were no significant differences between the specific variables with respect to the gender of the participating teachers in the experimental group (all *p* > 0.05).

### 3.2 Differences between pre-and post-programme AQ-27 scores in the experimental group

The results showed that the differences between AQ-27 scores between pre-programme (T0) and post-programme (T1) were statistically significant in all dimensions of the scale (Pity, Anger, Danger, Fear, Coercion, Segregation and Avoidance) (*p* < 0.001), except for Responsibility and Helping (both *p* > 0.05) (Table 4).

**Table 4:** Differences between AQ-27 scores in T0 and T1 in the experimental group.

AQ-27 dimension	Range	Pre-program (T0) Mean (SD ^†^)	Post-program (T1) Mean (SD ^†^)	*p-value*	95% CI^‡^	
					Lower	Higher
Responsibility	3–27	8.65 (3.40)	8.55 (3.97)	0.753	−0.51	0.70
Pity	3–27	18.69 (4.53)	16.66 (3.99)	0.000**	0.94	3.11
Anger	3–27	8.61 (4.03)	6.70 (3.92)	0.000**	1.08	2.74
Danger	3–27	12.03 (5.47)	7.98 (4.52)	0.000**	2.92	5.06
Fear	3–27	10.38 (5.79)	6.83 (4.59)	0.000**	2.40	4.70
Help	3–27	22.08 (3.68)	21.37 (4.61)	0.146	−0.25	1.66
Coercion	3–27	19.79 (4.71)	16.13 (5.79)	0.000**	2.41	4.90
Segregation	3–27	9.51 (4.91)	6.79 (4.32)	0.000**	1.74	3.70
Avoidance	3–27	13.91 (5.28)	11.73 (6.06)	0.003**	−1.14	3.56

T-Student test application.

SD^†^, standard deviation; CI^‡^, Confidence Interval.

** *p*-value <0.01.

The results on the AQ-27 pre-post programme scale of the experimental group, taking gender into account, showed statistically significant differences in the scores on the dimensions of Pity and Anger (*p* < 0.05), Danger, Fear, Coercion and Segregation (*p* < 0.01) for the male group. In the female group, statistically significant differences were obtained for the dimensions of Pity, Anger, Danger, Fear, Coercion, Segregation and also for Avoidance (all *p* < 0.01) (Table 5).

**Table 5:** Gender differences in the AQ-27 score in T0-T1 in the experimental group.

AQ-27 dimension	Range	Pre-program (T0) Mean (SD ^†^)	Post-program (T1) Mean (SD ^†^)	*p-value*	95% CI	
					Lower	Higher
Men						
Responsibility	3–27	8.19 (2.89)	7.84 (2.77)	0.482	−0.96	3.42
Pity	3–27	18.34 (4.91)	16.47 (3.95)	0.033*	0.40	7.13
Anger	3–27	9.03 (3.65)	7.09 (4.35)	0.034*	−1.55	4.63
Danger	3–27	11.59 (4.41)	7.94 (4.07)	0.001**	−0.17	7.10
Fear	3–27	9.94 (4.66)	6.75 (3.85)	0.002**	−0.89	5.05
Help	3–27	22.22 (2.76)	21.66 (3.58)	0.394	−3.00	7.61
Coercion	3–27	19.41 (3.99)	15.63 (5.21)	0.002**	−3.39	6.62
Segregation	3–27	9.91 (4.35)	7.25 (4.15)	0.002**	−9.05	−1.71
Avoidance	3–27	13.22 (4.22)	11.84 (5.49)	0.273	−3.76	5.45
Women						
Responsibility	3–27	8.86 (3.61)	8.87 (4.39)	0.971	−0.80	1.17
Pity	3–27	18.85 (4.37)	16.75 (4.04)	0.004**	0.45	3.64
Anger	3–27	8.42 (4.23)	6.52 (3.73)	0.000**	0.85	3.32
Danger	3–27	12.23 (5.91)	8.00 (4.73)	0.000**	2.78	6.10
Fear	3–27	10.58 (6.26)	6.86 (4.91)	0.000**	2.48	6.12
Help	3–27	22.01 (4.04)	21.24 (5.02)	0.231	−1.24	2.22
Coercion	3–27	19.96 (5.02)	16.35 (6.06)	0.000**	1.57	5.03
Segregation	3–27	9.34 (5.16)	6.58 (4.40)	0.000**	1.41	4.72
Avoidance	3–27	14.23 (5.70)	11.68 (6.33)	0.006**	−0.35	4.30

T-Student test application.

SD^†^, standard deviation.

*p-value <0.05.

** *p*-value <0.01.

### 3.3 Difference between the AQ-27 pre-programme and three-month follow-up scores in the experimental group

The results showed that teachers who could be followed up at 3 months (*n* = 38) had statistically significant differences in most dimensions of the AQ-27, in such a way that the scores on Pity, Anger, Danger, Fear, Help, Coercion and Segregation were statistically lower at T2 (*p* < 0.01). In addition, although the changes were not statistically significant in Responsibility and Avoidance, scores also decreased at T2 (Table 6).

**Table 6:** Differences in the AQ-27 scores at T0 and T2 in the experimental group.

AQ-27 dimension	Range	Pre-program (T0) Mean (SD ^†^)	Post-program (T2) Mean (SD ^†^)	*p-value*	95% CI	
					Lower	Higher
Responsibility	3–27	10.08 (3.59)	9.71 (4.26)	0.547	−0.86	1.59
Pity	3–27	19.29 (4.28)	16.37 (3.90)	0.003**	1.09	4.74
Anger	3–27	10.82 (4.28)	8.39 (5.08)	0.003**	0.85	3.98
Danger	3–27	14.08 (5.61)	10.11 (5.24)	0.000**	2.18	5.76
Fear	3–27	12.50 (6.40)	8.74 (5.73)	0.000**	1.90	5.62
Help	3–27	21.08 (3.71)	19.00 (6.23)	0.037**	0.13	4.02
Coercion	3–27	20.92 (4.64)	15.61 (6.73)	0.000**	1.87	6.49
Segregation	3–27	11.42 (4.59)	8.13 (4.99)	0.000**	−9.40	−5.38
Avoidance	3–27	15.53 (4.49)	14.32 (5.97)	0.304	−1.14	3.56

T-Student test application.

SD ^†^, standard deviation.

** *p*-value <0.01.

### 3.4 Difference in AQ-27 pre-programme and three-month follow-up scores in the control group

The results showed that there were no significant changes between the AQ-27 scores between pre-programme (T0) and 3 months (T2) for Responsibility (*p* = 0.906), Pity (*p* = 0.941), Anger (*p* = 0.552), Danger (*p* = 0.344), Fear (*p* = 0.804), Help (*p* = 0.414), Coercion (*p* = 0.536), Segregation (*p* = 0.030) y Avoidance (*p* = 0.174). However, a statistically significant reduction in the Segregation score (*p* < 0.05) was observed at T2.

### 3.5 Effect size of the anti-stigma training programme between pre-programme time and three-month follow-up (T0-T2) in the experimental vs. control group

The results showed that there were significant differences between the experimental group and the control group in the level of stigmatisation from pre-programme time (T0) to follow-up (T2), in such a way that the mean scores on the AQ-27 scale of the experimental group were significantly lower than those of the control group in the dimensions of Pity, Coercion and Segregation with a moderate to large effect size (all *d* ≥ 0.50) (Table 7).

**Table 7:** Size of Cohen’s d effect.

AQ-27 dimension	Group	Mean (SD)	T-Student	*p-value*	ES ^§^
Responsibility	Exp^†^	9.71 (4.26)	0.824	0.413	0.20
	Con^‡^	8.91 (3.87)			
Pity	Exp^†^	16.37 (3.90)	3.081	0.003*	0.72
	Con^‡^	19.30 (4.11)			
Anger	Exp^†^	8.39 (5.08)	0.550	0.584	0.11
	Con^‡^	7.82 (3.45)			
Danger	Exp^†^	10.11 (5.24)	−1.715	0.091	0.40
	Con^‡^	12.12 (4.55)			
Fear	Exp^†^	8.74 (5.73)	−0.329	0.743	0.08
	Con^‡^	9.12 (3.74)			
Help	Exp^†^	19 (6.23)	−1.523	0.132	0.36
	Con^‡^	21.03 (4.77)			
Coercion	Exp^†^	15.61 (6.73)	−2.102	0.039*	0.50
	Con^‡^	18.64 (5.17)			
Segregation	Exp^†^	8.13 (4.99)	−2.725	0.008*	0.65
	Con^‡^	11.39 (5.74)			
Avoidance	Exp^†^	14.32 (5.97)	1.876	0.065	0.45
	Con^‡^	11.94 (4.45)			

95% wald CI. Exp^†^, experimental group; Con^‡^, control group; ES^§^, effect size with Cohen’s d.

*p-value<0.05.

## 4 Discussion

The present study aims to provide evidence on the effectiveness of an intervention based on a training programme in the prevention and promotion of mental health, carried out by a specialised mental health nurse, with the aim of reducing stigmatising beliefs and attitudes of teachers working with the adolescent population, and to test whether this effectiveness is maintained in the long term in several different high schools in Spain.

To achieve this goal and address some of the limitations observed in the literature, this research includes two significant improvements compared to previous studies (16, 25, 29, 42), the inclusion of two more training modules in the programme (emotions and community resources for addressing mental health problems) and the implementation of a longitudinal evaluation.

Thus, in general, our results have shown that after the anti-stigma training programme, teachers showed that they had fewer stigmatising beliefs related to mental health problems; this attitude, moreover, was maintained over time. This reduction in stigmatising attitudes occurred especially with stereotypes and prejudices related to the feeling of pity towards people with mental health problems, the belief that these people should be separated from their environment and the conviction that it is necessary to decide for them and force them to receive treatment.

When interpreting our results, it is worth taking into account the high level of stigmatisation found in all participating teachers who showed a predominance of attitudes such as believing that people with psychopathology should be forced to receive medical treatment (Coercion) or the belief that they were not capable of living autonomously and independently and therefore needed more help than anyone else (Help). Similarly, most teachers reported feeling sorry for them (Pity), believing that they were dangerous people (Danger) and should therefore be avoided (Avoidance). However, beliefs that people with mental health disorders are responsible for their psychopathology (Responsibility), the belief that these people should be separated from the rest of the population (Segregation) or feelings of anger and fear associated with these people were scarce. These results are similar to those found in the previous pilot study conducted by the research team (29) and other studies such as that of Chaves A et al. (43), which measured attributional stigma related to Obsessive-Compulsive Disorder in teachers in Spain. The findings are also similar to those from studies conducted with other populations such as university students in the health sciences (44–47).

These findings might show that despite recommendations to apply a biopsychosocial and inclusive model in the Spanish educational and socio-health context in the care of health problems (48) the biomedical model and pharmacological prioritisation still prevail (44, 46). This would mean that after almost 50 years of research, the concept of mental illness defined in neurophysiological parameters (49) remains entrenched in the general population, which, together with the widespread belief that the origin of mental health problems is mostly genetic and therefore unavoidable (50) would lead to more paternalistic and less enabling views towards the person suffering from a mental disorder (51).

Regarding the differences in the level of stigmatisation obtained by the participating teachers before and after the training programme, there was a decrease in all their stigmatising attitudes, except for the belief that people with a mental disorder are responsible for it and that they cannot manage their illness (Responsibility) and the feeling of pity towards them (Pity), results that are in line with the findings of the pilot study of this research (29). However, if we review the structural characteristics in the validation process of the AQ-27 scale to the Spanish language, its authors recommend conducting complementary studies to analyse these stigmatising attitudes in greater detail given that they showed limited internal consistency, which could be explained by cross-cultural and language differences (41). Furthermore, special caution should be shown when comparing these results with those of the scientific literature, given that the scores obtained in some stigmatising attitudes – more specifically Pity and Help – could be misinterpreted. For example, there are studies that, by obtaining high scores in Pity and Helpfulness, have concluded that their participants were more compassionate and more willing to offer help to a person with a mental disorder (43, 46, 52); however, the AQ-27 scale is a negative scale, so the higher the score on an item, the higher the stigma attribution.

With regard to the differences in stigmatising attitudes between men and women, it can be seen that in both cases the presence of stigmatising beliefs is similar before the programme, coinciding with the findings in Saguem et al. study (50). Even though the level of stigma amongst both men and women decreased after completing the training programme, it is important to note that women maintained the beliefs that the person with a mental disorder is not responsible and cannot take care of it and therefore needs help and assistance to manage it. In the case of men, the avoidance attitude towards people with this type of illness is added. This observation coincides with other research concluding that women are more knowledgeable, less socially stigmatised and more willing to offer help (46, 52, 53).

In our study, the fact that women were more likely than men not to avoid but rather accompany people with mental health problems could be related to the assumption of gender stereotypes in which women tend to assume the role of caregivers. In fact, in Spain, studies show that women are the main providers of informal care due to a cultural legacy based on the patriarchy (54).

In this same line of results, it should be added that in our study no differences were found between males and females when reporting their experience of contact with students in need of specialised mental health medical help or showing self-harm. Although these responses would be in line with Manjula et al. results (55), this is particularly alarming as it is inconsistent with the high numbers of adolescents receiving treatment for mental health problems or self-harm, which are reported to be around 20% by national and international agencies (1, 2).

In our study, these results could mean that teachers are not able to detect or accompany students with mental health problems, a fact that could be related to the levels of stigma presented by teachers prior to the training programme (46). Hence authors such as Granada-López et al. (18), Sibanda et al. (56) and Imran et al. (57), all highlight the urgent need for increased teacher training in mental health in the classroom. Further research should not only explore whether teachers intervene and follow up with students who have a mental health problem, but also how and under what conditions this intervention is carried out (17).

As for the longitudinal results obtained in our study, they suggest that the anti-stigma training programme had a positive effect on the level of teacher stigma in the long term as well, since all the negative beliefs assessed were reduced 3 months after the end of the programme. This was especially so with attitudes that showed higher levels of stigma before starting the programme, such as feeling sorry for a person with a mental disorder (Pity), forcing them to receive medical treatment (Coercion) and even considering their separation from the rest of the population (Segregation). However, beliefs related to responsibility for the mental disorder and avoidance of people with psychopathology did not disappear so easily, something which should be addressed in depth in future research.

Although these findings may be biassed by one of the main limitations of the study—the loss or lack of cooperation of participants during the intervention phase—such circumstances are common in follow-up studies (69, 70). Additionally, it is important to consider that the high dropout rates could be intrinsically linked to the current state of public employment in the non-university teaching sector. According to the database of the Register of Teaching Staff, in Spain, for the 2022/23 academic year, it was estimated that approximately 63,870 teachers were in temporary employment (58), which may contribute to excessive turnover of teaching staff each academic cycle, thereby fostering employment volatility and, potentially, higher attrition rate. Future research should consider these particularities, as it would be of interest to determine the long-term effect of the programme (e.g., 6 months or 1 year). In any case, it is important to recognise the efforts made to implement this anti-stigma training programme. As McCullock and Scrivano (42) report, to date, the scientific evidence supporting the validity of practical interventions aimed at eradicating stigma remains limited.

Thus, the good results obtained in this research could be explained, at least in part, by several different aspects. First, the anti-stigma programme was implemented entirely by a specialised mental health nurse, who is considered a health professional trained to provide interventions related to increasing mental health literacy and reducing stigma in different populations (26, 28, 59). Secondly, the relevance of the topics addressed, the content included in the programme, and its focus on promoting identification and reflection on the impact of the media on the perception of mental health disorders in society is highlighted (60). This last aspect could be crucial in the learning process, as it seems that stigma has a significant cultural and media influence (14). Moreover, actual testimonies were also included as an effective strategy to combat stigma (52, 61). We believe that the inclusion of these practises into the training programme was a good decision, especially given Imran et al. (57) recommendations, which highlight the importance of including anti-stigma measures in any mental health training programme. These actions could prevent the negative consequences of stigma for students with mental health problems, such as social isolation and being deprived of help (62).

Furthermore, informational content on community support services and on the referral procedure to health services was included, following the recommendations of Granada-López et al. (18) and Arsland and Karabey (16). The latter emphasised the relevance of presenting and learning about primary care facilities as one of the main access points to health care for minors, given the lack of knowledge amongst teachers and students (16). These additions to the training programme contributed to improving teacher skills, ensuring appropriate follow-up and referral, and reducing stereotypes and prejudices associated with mental health.

Finally, it is essential to highlight the fact that the training programme focused on expanding knowledge and correcting misconceptions about mental health, as well as providing teachers with the appropriate tools and materials to address the issue with adolescents in the classroom, as teaching mental health to students, led by their teachers, seems to have a positive impact on both (17).

It would therefore be a good idea for teachers to incorporate this type of training not only in their teaching career, as Al Omari et al. (14) suggest, but also as part of their university education (18). In fact, and according to Gallego et al. (63) research, teacher training students in Spain were found to exhibit high levels of mental health stigma compared to other countries.

From our findings, we not only endorse this suggestion, but also recommend that this training be extended to the whole educational community (64), and further, involve students (56) and their families, especially in areas with social inclusion difficulties or limited resources, as noted by Praharso, Pols and Tiliopoulos (65) and Javed et al. (66). Whilst authors such as Nalipay and Simon (67) advise brief, targeted training in the case of recent incidents, we would propose carrying out this programme on a regular basis and with booster sessions over time.

In summary, we agree with other research that short training programmes are beneficial in addressing mental health in the educational setting (42, 55), that the specialised mental health nurse plays a key role in these programmes (14, 28), and that these should form part of teachers’ compulsory curricular subjects (12).

### 4.1 Limitations

Firstly, it is important to recognize the existence of a possible selection bias, due to the inherent characteristics of the sample. This was because participation did not only depend on the volunteerism of the participants. Rather, it was also not possible to perform a randomisation that would guarantee an adequate sample size in the experimental group due to logistical factors and limited resources.

Secondly, in relation to the above, it is worth considering the dropout rate in the follow-up period of our study, which, although similar and even lower than in other studies, such as Manjula et al. (55), reached 58%. This reduction in sample size may have conditioned the results related to the effect size of the training programme and should be interpreted with caution.

Thirdly, regarding the instrument used to measure the level of stigma, and taking into account that the participants were aware of the goals of the study, social desirability bias, which can occur in the use of self-administered scales, may have been present. In addition, the AQ-27 scale used in this study (despite its being validated for use in the Spanish-speaking population), presents some particularities that should be taken into account when drawing conclusions regarding the score. For example, it presents a low consistency in some dimensions such as Responsibility and Pity and its interpretation differs in the peer-reviewed literature. In this regard, future research should include a scale that analyses stigma towards mental disorders in general and also takes into account the cultural factor (14, 44).

## 5 Conclusion

The findings provide preliminary evidence that the anti-stigma training programme conducted had a positive impact on reducing the level of stigma attribution amongst teachers by obtaining a significant decrease in stigmatising beliefs at the end of the programme. It is noted that teachers showed a reduction in feelings of Pity, Anger and Fear towards people with a mental disorder, as well as a reduction in the perception of Danger and the need to avoid or segregate them from society (Avoidance and Segregation). Even a decrease in the belief that these people should be forced to receive medical treatment (Coercion) was observed.

Similarly, the reduction in prejudice and stereotypes was maintained in the long term, especially in those attitudes related to Pity, Coercion to receive treatment and Segregation from their environment.

Although future research could strengthen the contents of the programme in order to obtain more solid results, the appropriateness of the contents and the fundamental role of the specialised mental health nurse who led the training programme should be highlighted. The possibility of comparing these results to a control group increases the robustness of the research and provides greater confidence in the conclusions drawn.

Finally, it should be noted that, despite the limitations, this is the first study to show ground-breaking results on the evaluation of the long-term effectiveness of a training programme aimed at reducing attributional stigma associated with mental health in high school teachers.

## Data Availability

The original contributions presented in the study are included in the article/Supplementary material, further inquiries can be directed to the corresponding author.
