# Supplementary material for: Working on mental health stigma in education: a multicentre community-based clinical trial
Source: Front Public Health. 2025 Jun 27;13:1515444. doi: 10.3389/fpubh.2025.1515444 (PMC12248066; doi:10.3389/fpubh.2025.1515444)
Supplement: Supplementary file 1 [file Table_1.docx]

Supplementary Material

Supplementary table 1. Differences between the Experimental Group and the Control Group, in the specific variables related to the experience of the teaching staff and the mental health of their students, according to sex.

| **Variables** | **Experimental group** | | **Control group** | | **Total** | ***p*-valor^1^** |
| --- | --- | --- | --- | --- | --- | --- |
|  | **Woman (AF)** | **Male (AF)** | **Woman (AF)** | **Male (AF)** |  |  |
| Tutoring |  | | | |  | 0,626 |
| Yes | 41 | 14 | 20 | 14 | 89 |  |
| No | 30 | 18 | 22 | 10 | 80 |  |
| Are you aware of any students who have needed specialised mental health help? |  | | | |  | 0,444 |
| Yes | 31 | 12 | 24 | 12 | 79 |  |
| No | 39 | 20 | 18 | 12 | 89 |  |
| Nc | 1 | 0 | 0 | 0 | 1 |  |
| Are you aware of any students who self-harm and/or have tried to self-harm? |  | | | |  | 0,960 |
| Yes | 28 | 12 | 24 | 14 | 78 |  |
| No | 43 | 20 | 18 | 10 | 91 |  |
| Are you aware of any students who have tried to take their own life? |  | | | |  | 0,382 |
| Yes | 34 | 17 | 16 | 12 | 79 |  |
| No | 36 | 15 | 26 | 12 | 89 |  |
| Nc | 1 | 0 | 0 | 0 | 1 |  |
| Have you had to act in the psychological crisis of a pupil? |  | | | |  | 0,796 |
| Yes | 39 | 14 | 22 | 14 | 89 |  |
| No | 32 | 18 | 20 | 8 | 78 |  |
| Nc | 0 | 0 | 0 | 2 | 2 |  |
| If so,  How did you feel during the intervention? |  | | | |  | 0,333 |
| Very well | 0 | 0 | 0 | 0 | 0 |  |
| OK | 1 | 0 | 0 | 0 | 1 |  |
| Normal | 5 | 2 | 2 | 0 | 9 |  |
| Bad | 17 | 6 | 6 | 6 | 35 |  |
| Very bad | 13 | 6 | 2 | 8 | 29 |  |
| Nc | 35 | 18 | 32 | 10 | 95 |  |

1 *p*-value obtained from the Chi-square test; AF: absolute frequency; RF: relative frequency; SD: Standard Deviation.

Nc^ⴕ^: No answer AF^‡^: Absolute frequency

**
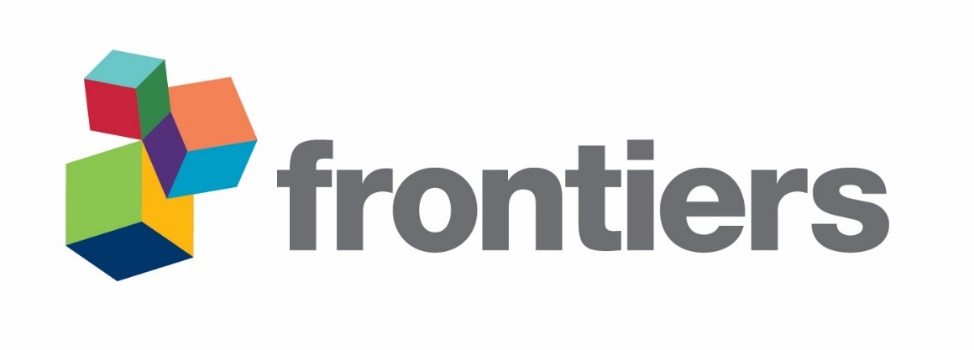
**
